# Metabolomic Analysis of *Lactobacillus acidophilus, L. gasseri, L. crispatus*, and *Lacticaseibacillus rhamnosus* Strains in the Presence of Pomegranate Extract

**DOI:** 10.3389/fmicb.2022.863228

**Published:** 2022-05-18

**Authors:** MaryClaire Chamberlain, Sarah O'Flaherty, Natalia Cobián, Rodolphe Barrangou

**Affiliations:** Department of Food, Bioprocessing and Nutrition Sciences, North Carolina State University, Raleigh, NC, United States

**Keywords:** metabolomics, *Lactobacillus*, probiotic, pomegranate extract, prebiotic

## Abstract

*Lactobacillus* species are prominent inhabitants of the human gastrointestinal tract that contribute to maintaining a balanced microbial environment that positively influences host health. These bacterial populations can be altered through use of probiotic supplements or *via* dietary changes which in turn affect the host health. Utilizing polyphenolic compounds to selectively stimulate the growth of commensal bacteria can have a positive effect on the host through the production of numerous metabolites that are biologically active. Four *Lactobacillus* strains were grown in the presence of pomegranate (POM) extract. Two strains, namely, *L. acidophilus* NCFM and *L. rhamnosus* GG, are commonly used probiotics, while the other two strains, namely, *L. crispatus* NCK1351 and *L. gasseri* NCK1342, exhibit probiotic potential. To compare and contrast the impact of POM on the strains' metabolic capacity, we investigated the growth of the strains with and without the presence of POM and identified their carbohydrate utilization and enzyme activity profiles. To further investigate the differences between strains, an untargeted metabolomic approach was utilized to quantitatively and qualitatively define the metabolite profiles of these strains. Several metabolites were produced significantly and/or exclusively in some of the strains, including mevalonate, glutamine, 5-aminoimidazole-4-carboxamide, phenyllactate, and fumarate. The production of numerous discrete compounds illustrates the unique characteristics of and diversity between strains. Unraveling these differences is essential to understand the probiotic function and help inform strain selection for commercial product formulation.

## Introduction

The human gastrointestinal tract (GIT) harbors one of the most densely populated bacterial communities on Earth, which is highly diverse and complex (Holscher, 2017). These microorganisms form a symbiotic relationship with the host as they metabolize undigested food remnants and transform them into compounds that can be used as energy by the host or contribute to host health. *Lactobacillus* species are prominent microorganisms that help maintain a balanced microbiota within human microbiome habitats such as the oral cavity, gastrointestinal tract, and vaginal tract (Huttenhower et al., 2012). A healthy gut environment can be improved with probiotic supplementation, which is defined by the International Scientific Association for Probiotics and Prebiotics (ISAPP) as “live microorganisms that, when administered in adequate amounts, confer a health benefit to the host”. These effective probiotic strains are transient residents of the host, as they can survive at low pH and high bile concentration environments in the GIT, adhere to intestinal epithelial cells, and inhibit the growth of pathogenic microorganisms (Blum et al., 1999; Likotrafiti and Rhoades, 2016; Somashekaraiah et al., 2019).

In addition to probiotic consumption, the GIT microbiota can be altered through human diets. Prebiotics are substrates that are selectively utilized by host microorganisms to confer a health benefit (Gibson et al., 2017), and traditionally, they consist of non-digestible carbohydrates such as inulin, fructooligosaccharides, galactooligosaccharides, and human milk oligosaccharides. Research in the past decades has shifted to studying plant-derived polyphenolic compounds as potential prebiotics due to their beneficial biological effects. Polyphenols are phytochemicals that are native in plants to protect them from environmental stresses, and these compounds differ considerably in their carbon structure, hydroxylation patterns, and glycosylation and acylation of the heterocyclic rings, which impacts their bioavailability in humans (Scalbert and Williamson, 2000). The polyphenolic compounds that reach the colon to be degraded and depolymerized by microbes result in a variety of 1,3 diphenylpropanes, γ-valerolactones, benzoic acids, phenylacetic acids, phenylpropionic acids, and other aromatic compounds (Justesen et al., 2000; Gonthier et al., 2003; Rios et al., 2003; Urpi-Sarda et al., 2009).

The investigation of the human gut microbiome has progressed over time with advances in high-throughput sequencing technologies to further understand the relationship between the host and microbial population. The most recent emerging field of systems biology research is metabolomics, which provides a comprehensive analysis of all the metabolites involved in general metabolic reactions necessary for the maintenance, growth, and normal functions of a cell (Dettmer et al., 2007). Utilizing metabolomic technology as a tool to study the gut metabolome offers valuable information regarding metabolic pathways, health of the environment, and responses to internal or external perturbations (Bernini et al., 2009; Cambeiro-Perez et al., 2018, 2020).

Metabolomic analysis of *Lactobacillus* species has gained attention from the food industry due to their use as starter cultures in fermented foods. Studies have observed the metabolite changes during fermentation and analyzed the compounds that contribute to organoleptic properties or bio-preservative effects in fermented vegetables (Park et al., 2016; Zhao et al., 2016), dairy (Piras et al., 2013; Palomo et al., 2014), soy (Namgung et al., 2010; Kim et al., 2012), and beverages (Lee et al., 2009; López-Rituerto et al., 2012). Due to the important role of lactobacilli in human health, researchers have utilized metabolomics in human clinical studies to identify the effects of probiotic treatments (Hong et al., 2011; Ghini et al., 2020). There have been numerous *in vivo* and *in vitro* studies to analyze the effects of traditional prebiotics, such as inulin-type fructans (Vandeputte et al., 2017), galactooligosaccharides (Cheng et al., 2018), and xylooligosaccharides (Yang et al., 2019), with probiotics, whereas the use of polyphenolic compounds in combination with probiotics has not been studied as extensively. Pomegranates are rich in polyphenols, specifically punicalagins, punicalins, and ellagic acid, which are part of a group of hydrolyzable tannins designated as ellagitannins (Garcia-Muñoz and Vaillant, 2014). The evidence for pomegranates to have a prebiotic effect still needs to be substantiated by clinical studies; however, *in vitro* studies have shown to promote the growth of beneficial bacteria and produce smaller metabolites, such as urolithins (Bialonska et al., 2009, 2010). Identifying bacterial fermentation products from various substrates contributes to understanding their beneficial effects on the host. In this study, we used an untargeted metabolomic approach to determine how pomegranate extract influenced the growth and biotransformation abilities of four *Lactobacillus* strains. We analyzed two commonly used probiotics (*L. acidophilus* NCFM and *L. rhamnosus* GG) and two potential probiotics that are found within the human GIT (*L. crispatus* NCK1351 and *L. gasseri* NCK1342). This technique allowed for a qualitative measurement of each of the metabolites produced. We aimed to investigate the metabolic profiles of each *Lactobacillus* strain and compare and contrast the metabolites and associated pathways among them.

## Materials and Methods

### Pomegranate (POM) Extract Preparations

The pomegranate extract was composed of POMx spray-dried pomegranate powder from POM Wonderful, Inc., Los Angeles, CA (Heber et al., 2007). The preparation of the POM extract for the bacterial growth curves was followed as described earlier (Henning et al., 2017). POM extract was prepared by dissolving 7 mg/ml of POM extract in ultrapure water and vortexed for 10 min. The stock solution was then centrifuged at 3,226 × *g* for 10 min, and the supernatant was filtered with a 0.45-μm membrane filter and stored in aliquots at −20°C. Semi-defined media (SDM) was used as the base media (Kimmel and Roberts, 1998) for the growth curves and with added glucose (1% for transfers or 0.5% for growth assay). A previous study reported that 400 μg/ml of POMx stimulated the growth of bifidobacteria and lactobacilli while inhibiting the growth of the *Enterobacteriaceae, Clostridia*, and *Bacteroides fragilis* groups (Li et al., 2015). In this study, the stock POM extract was diluted in SDM to 400 μg/ml for the transfers and the growth assays.

### Growth Assays

The details of the *Lactobacillus* strains are summarized in Table 1. *Lactobacillus acidophilus* NCFM, *Lacticaseibacillus rhamnosus* GG, *Lactobacillus crispatus* NCK1351, and *Lactobacillus gasseri* NCK1342 were inoculated from −80°C glycerol stocks into de Man-Rogosa-Sharpe (MRS) broth (Difco Laboratories, Inc., Detroit, MI) and grown overnight in a water bath at 37°C. Overnight cultures were inoculated 1% v/v into SDM (1% glucose, control) and SDM (1% glucose with 400 μg/ml POM extract) and grown overnight in a water bath at 37°C for an additional two transfers. After the second transfer, overnight cultures were then inoculated 1% v/v into SDM (0.5% glucose, control) and SDM (0.5% glucose with 400 μg/ml POM extract) and grown in a water bath at 37°C. Optical density (600 nm) was measured with a Biowave Cell Density Meter CO8000 (Biochrom Ltd, Cambridge, England) for up to 24 h. Simultaneously, at 0, 4, 8, 12, and 24 h cultures were plated on MRS agar medium and incubated anaerobically at 37°C for 48 h before colonies were counted.

**Table 1:** Strains used in this study: information regarding *Lactobacillus* strain type, origin, and accession number.

**Genera**	**Species**	**Strain**	**Source**	**Origin**	**Genome Accession Number**
*Lactobacillus*	*acidophilus*	NCFM	NCSU^a^	Commercial (human)	GCA_000011985.1
*Lactobacillus*	*gasseri*	NCK1342	NCSU	Patient endoscopy	PQWW00000000.1
*Lactobacillus*	*crispatus*	NCK1351	NCSU	Healthy endoscopy	GCA_008694745.1
*Lacticaseibacillus*	*rhamnosus*	GG	DuPont	Commercial (human)	GCA_000026505.1

a *North Carolina State University*.

### API Assays

The carbohydrate utilization and enzyme activity were assayed with Analytical Profile Index (API) tests (Biomerieux, Durham, NC) in duplicate. For the carbohydrate utilization, cultures were grown overnight in MRS broth at 37°C in a water bath; 1 ml of culture was then centrifuged at 8,609 × *g* for 1 min at room temperature. The cells were washed twice with phosphate-buffered saline (PBS) and then resuspended with 1 ml PBS. Cells (2% v/v) were added to 10 ml PBS, and the absorbance (Abs) at 600 nm was measured. Based on the Abs reading, the volume of the cell suspension needed to achieve ca. Abs 0.05 was calculated. Following the manufacturer's instructions, the calculated cell suspension was added to the API 50 CHL medium and inverted several times. The API tray wells were inoculated with the cell suspension and CHL medium and covered with mineral oil. Trays were incubated at 37°C under ambient atmospheric conditions, and results were recorded at 24 and 48 h.

For the enzyme activity assay, cultures were grown overnight in MRS broth at 37°C in a water bath. Cells were washed in ultrapure distilled water (1:1 v/v) and centrifuged for 5 min at 3,226 × *g*. Cells achieved a turbidity of 5–6 McFarland, and 65 μl was administered to each cupule. Trays were incubated at 37°C under ambient atmospheric conditions. After incubation, one drop of ZYM A and ZYM B reagents was added to each cupule and placed 10 cm under a light source (1,000 W bulb) for 10 s. Results were recorded 5 min after the 10-s light exposure according to the manufacturer's instructions.

### Sample Preparation for Metabolomic Analysis

Cultures were grown and transferred as described earlier for the *Lactobacillus* growth assays. Following two transfers in SDM (1% glucose with 400 μg/ml POM extract), cells were inoculated 1% v/v into 10 ml of SDM (0.5% glucose with 400 μg/ml POM extract) and grown for 16 h at 37°C in a water bath. After 16 h, the cells were centrifuged at 3,226 × *g* for 10 min, and 200 μl of cell-free supernatant was transferred to labeled cryogenic tubes for immediate storage at −80°C. Controls consisted of SDM media with no POM extract, SDM media with POM extract (400 μg/ml) at 0 h of incubation, and SDM media with POM extract (400 μg/ml) at 16 h of incubation. Quadruplicate cell-free supernatant samples were shipped on dry ice to Metabolon Inc. (Durham, NC) for analysis.

### Untargeted Metabolite Analysis

Global metabolite profiles were obtained for each sample by a commercial laboratory (Metabolon, Inc.). Additional standards were purchased and added to the Metabolon library, including 8-O-methyl urolithin A, gallic acid, punicalagin, and punicalin (Sigma-Aldrich, St. Louis, MO). Each sample received was accessioned into the Metabolon Laboratory Information Management System (LIMS) and was assigned by the LIMS a unique identifier that was associated with the original source identifier only. Samples were prepared using the MicroLab STAR^®^ automated system (Hamilton Company, Reno, NE).

Several recovery standards were added prior to the first step in the extraction process for QC purposes. To remove protein, dissociate small molecules bound to protein or trapped in the precipitated protein matrix, and to recover chemically diverse metabolites, proteins were precipitated with methanol under vigorous shaking for 2 min (GenoGrinder 2000, Glen Mills Inc, NJ) followed by centrifugation. The resulting extract was divided into five fractions: two for analysis by two separate reverse-phase (RP)/UPLC-MS/MS methods with positive ion mode electrospray ionization (ESI), one for analysis by RP/UPLC-MS/MS with negative ion mode ESI, one for analysis by HILIC/UPLC-MS/MS with negative ion mode ESI, and one sample was reserved for backup. Samples were placed briefly on a TurboVap^®^ (Zymark) to remove the organic solvent. The sample extracts were stored overnight under nitrogen before preparation for analysis.

All methods utilized a Waters ACQUITY ultra-performance liquid chromatography (UPLC) and a Thermo Scientific Q-Exactive high-resolution/accurate mass spectrometer interfaced with a heated electrospray ionization (HESI-II) source and Orbitrap mass analyzer operated at 35,000 mass resolution. The sample extract was dried and then reconstituted in solvents compatible with each of the four methods. Each reconstitution solvent contained a series of standards at fixed concentrations to ensure injection and chromatographic consistency. One aliquot was analyzed using acidic positive ion conditions, chromatographically optimized for more hydrophilic compounds. In this method, the extract was gradient eluted from a C18 column (Waters UPLC BEH C18-2.1 × 100 mm, 1.7 μm) using water and methanol, containing 0.05% perfluoropentanoic acid (PFPA) and 0.1% formic acid (FA). Another aliquot was also analyzed using acidic positive ion conditions; however, it was chromatographically optimized for more hydrophobic compounds. In this method, the extract was gradient eluted from the same aforementioned C18 column using methanol, acetonitrile, water, 0.05% PFPA, and 0.01% FA and was operated at an overall higher organic content. Another aliquot was analyzed using basic negative ion optimized conditions using a separate dedicated C18 column. The basic extracts were gradient eluted from the column using methanol and water, however, with 6.5 mM ammonium bicarbonate, pH 8. The fourth aliquot was analyzed *via* negative ionization following elution from a HILIC column (Waters UPLC BEH Amide 2.1 × 150 mm, 1.7 μm) using a gradient consisting of water and acetonitrile with 10 mM ammonium formate, pH 10.8. The MS analysis alternated between MS and data-dependent MS^n^ scans using dynamic exclusion. The scan range varied slightly between methods but covered 70–1,000 m/z.

### Bioinformatics and Statistical Analysis

The raw data were extracted, peak-identified, and QC processed using Metabolon's proprietary hardware and software and curated using a variety of procedures to ensure that a high-quality data set was made available for statistical analysis and interpretation of the compounds (Lawton et al., 2008; Dehaven et al., 2010; Evans et al., 2012). The informatics system consisted of four major components, namely, the LIMS, the data extraction and peak-identification software, data processing tools for QC and compound identification, and a collection of information interpretation and visualization tools for use by data analysts. The hardware and software foundations for these informatics components were the LAN backbone and a database server running Oracle 10.2.0.1 Enterprise Edition.

Compounds from the metabolic profiling were semi-quantified using the area under the curve. The data were normalized and scaled to the median value for each compound. Following log transformation and imputation of missing values, if any, a Welch's two-sample *t*-test of the minimum observed value for each compound was performed in ArrayStudio. The two-sample *t*-test was used to identify biochemicals that differed significantly (*p* ≤ 0.05) between the samples and the control media with pomegranate at 16 h, and the resulting values were designated as the fold change values.

Rstudio v1.3. 1093-1 (http://www.rstudio.com/) was used for statistical analysis and graphs. Heat maps were generated with a hierarchical clustering model. The model used “cor()” and “hclust()” functions and a Spearman's rank-order correlation method. Principal component analysis (PCA) and partial least-squares discriminant analysis (PLS_DA) were made using MetaboAnalyst 5.0 (https://www.metaboanalyst.ca/). The boxplots were created by the “geom_boxplot()” and “geom_point()” functions. Apart from growth assays, graphs and boxplots were generated with ggplot2 in Rstudio.

## Results

### Growth Assays

**Figure 1:**
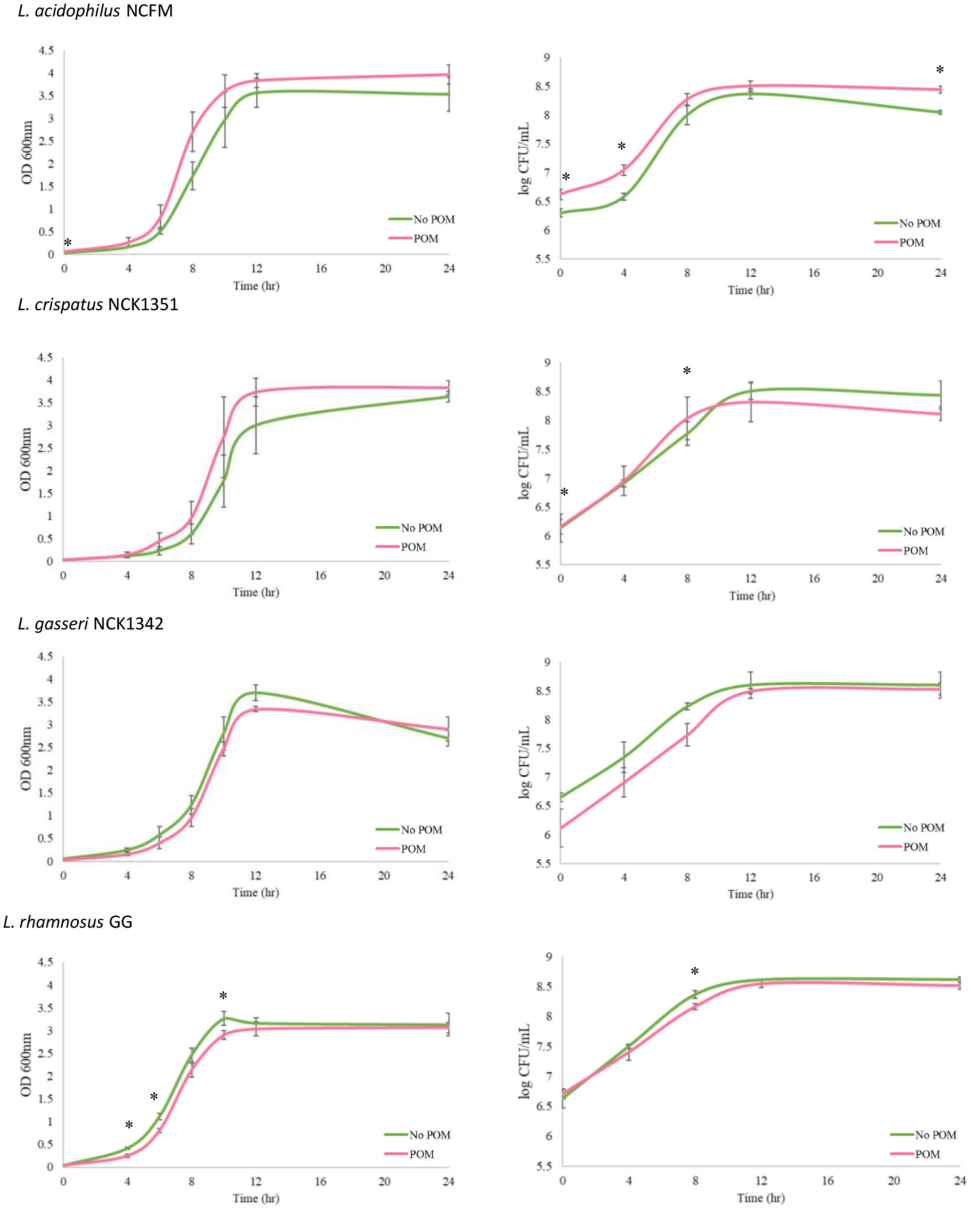
Growth of *Lactobacillus* strains with and without the POM extract (400 μg/ml). Growth assays were performed over a 24-h period with the *L. acidophilus* NCFM, *L. crispatus* NCK1351*, L. gasseri* NCK1342, and *L. rhamnosus* GG grown in SDM with 0.5% glucose and no POM extract (green curve, No POM) or in SDM with 0.5% glucose and 400 μg/ml of POM extract (red curve, POM). Graphs on the left-hand side show OD 600 nm values, and log CFU/ml growth curves are shown on the right-hand side. Error bars represent the standard deviation of three biological replicates, and * indicates *p* ≤ 0.05.

Growth assay was performed in SDM with a concentration of 0.5% w/v glucose, compared with the transfers that contained 1.0% w/v glucose. The decreased sugar content was conducted in order to provide the strains the opportunity to primarily utilize POM as a substrate. The OD 600 nm and CFU/ml counts were measured over 24 h for each strain grown in SDM with and without POM (400 μg/ml). In the case of OD measurements, there was no significant difference (*p* ≤ 0.05) for *L. acidophilus, L. crispatus*, and *L. gasseri* demonstrating POM extract did not have an inhibitory effect on growth. There was a minor but significant decrease in OD 600 nm readings at 4, 6, and 10 h for *L. rhamnosus*, but by 24 h there was no significant difference between the control and test group (Figure 1). Next, we measured the CFU/ml counts and determined the largest significant difference observed with *L. acidophilus* where the CFU/ml count increased in the media with POM extract at 24 h (Figure 1). Overall, each strain had similar growth patterns in SDM alone and SDM with POM, demonstrating that these strains are capable of growing in the presence of POM extract and potentially utilize the pomegranate as a substrate.

### API Assays

**Figure 2:**
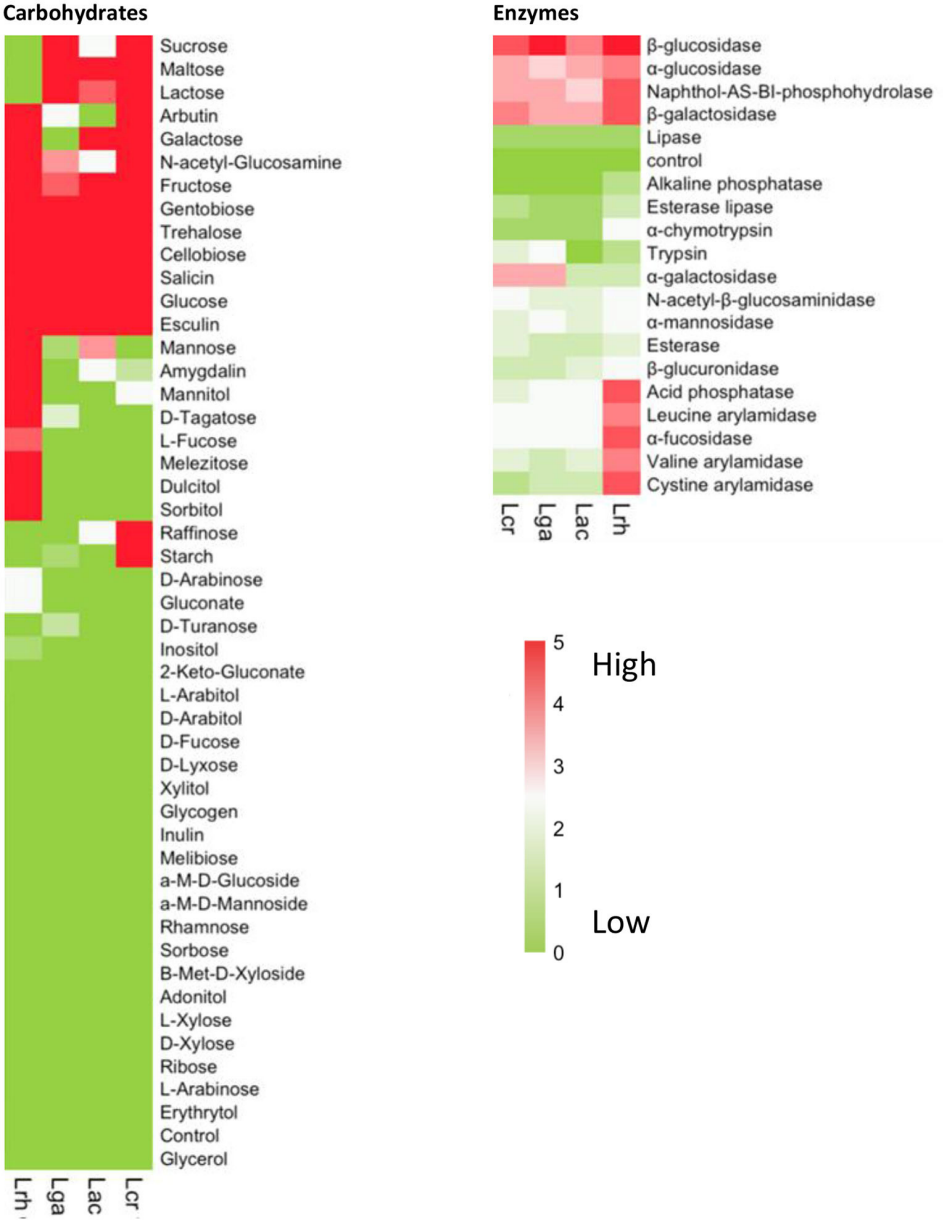
Carbohydrate and enzyme profiles of *Lactobacillus* strains. Carbohydrate utilization and enzymatic activity were determined using API assays for the four strains, namely, *L. acidophilus* NCFM (Lac), *L. crispatus* NCK1351 (Lcr), *L. gasseri* NCK1342 (Lga), and *L. rhamnosus* GG (Lrh). The heat maps show the mean values of two biological replicates with the values scored according to the manufacturer's instructions. The values range from 0 to 5 and are assigned to the standard color where zero represents no reaction and 5 represents a reaction of maximum intensity. The relative activity may be estimated from color strength.

We next determined the carbohydrate utilization and enzymatic activity profile for each of the four strains, namely, *L. acidophilus* NCFM (Lac), *L. crispatus* NCK1351 (Lcr)*, L. gasseri* NCK1342 (Lga), and *L. rhamnosus* GG (Lrh) (Figure 2). Results of the API assays determined that *L. rhamnosus* fermented the widest range of sugars, including sugar alcohols sorbitol, mannitol, and dulcitol, which were not fermented by the other three strains (Figure 2). One advantageous metabolic feature of lactobacilli is the ability to utilize lactose (Figure 2). In the case of *L. rhamnosus* GG, this feature is lost due to frameshifts in the antiterminator (*lacT*) and 6-phospho-β-galactosidase (*lacG*) genes (Kankainen et al., 2009). The inability of *L. rhamnosus* GG to ferment lactose was confirmed in the API assay (Figure 2). Additional genetic variations in enzymes or transporters also explain the inability of *L. rhamnosus GG* to utilize rhamnose, ribose, and maltose (Kankainen et al., 2009). The other three strains showed a similar carbohydrate utilization profile with *L. crispatus* being the sole strain that utilized both raffinose and starch (Figure 2).

Lactobacilli have many glycosyl hydrolases (GHs) that assist them in the metabolism of many types of mono-, di-, and polysaccharides, specifically α- and β-glucosidases and β-galactosidases. All four strains showed activity for these enzymes (Figure 2). Fucosyl-oligosaccharides are natural prebiotics that are found in intestinal mucin and human milk, and α-l-fucosidases have an important role in the cleavage of these compounds and have only been identified in a few *Lactobacillus* species (Rodríguez-Díaz et al., 2011), including *L. rhamnosus* GG (Morita et al., 2009). Our data showed that *L. rhamnosus* GG was the only one of the four strains with high α-l-fucosidases activity (Figure 2). As with the carbohydrate utilization assay, *L. rhamnosus* exhibited the widest enzymatic activity profile (Figure 2).

### Determination of Metabolite Profiles

#### Global Metabolomic Analysis of the Four *Lactobacillus* Strains

**Figure 3:**
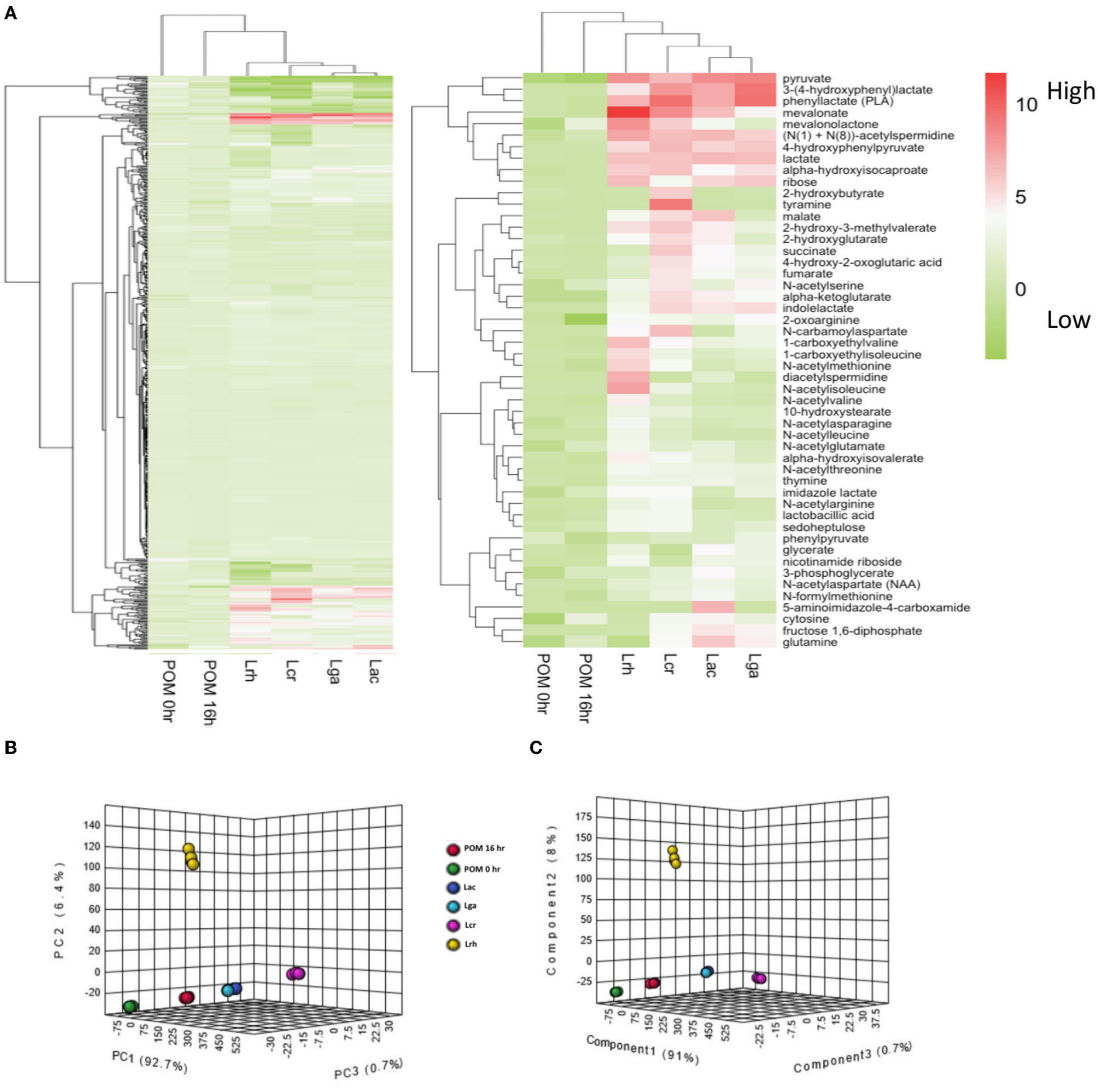
Global metabolomic analysis overview. **(A)** Hierarchical cluster analysis (HCA) of log2 transformed fold change values of the metabolomic data for the cell-free supernatants of the *Lactobacillus* strains and controls. The entire metabolite profile (left-hand side) and top 50 significant (*p* ≤ 0.05) highest produced metabolites (right-hand side) were performed for each strain. The legend shows the log2 transformed fold change values. **(B)** Principal component analysis (PCA) and **(C)** partial least-squares discriminant analysis (PLS_DA) of the global metabolite profile for the four *Lactobacillus* strains and controls. *L. acidophilus* NCFM (Lac), *L. crispatus* NCK1351 (Lcr)*, L. gasseri* NCK1342 (Lga), and *L. rhamnosus* GG (Lrh), SDM media with POM 400 μg/ml at T0 (POM 0 h) and T16 (POM 16 h).

To further elucidate which metabolites were produced by the strains when grown in the presence of POM, metabolomic analyses were performed on cell-free supernatants. Untargeted metabolite profiles of the samples from the four *Lactobacillus* strains grown in the presence of POM identified a total of 552 biochemicals, with 353 compounds confirmed with authentic standards. The number of metabolites that achieved statistical significance (*p* ≤ 0.05) with respect to the control media with no POM for *L. acidophilus, L. crispatus, L. rhamnosus*, and *L. gasseri* was 325, 325, 366, and 274, respectively. Carbohydrates are typically the preferred carbon source for lactobacilli; however, amino acids and other aromatic compounds, such as plant-derived polyphenols, can also serve as a carbon source to support bacterial growth. A hierarchical clustering analysis (HCA) of the fold change values of the entire metabolic profiles in the cell-free supernatants illustrates that *L. acidophilus* and *L. gasseri* were the most similar, followed by *L. crispatus*, and then *L. rhamnosus* (Figure 3A). Both the POM controls (0 and 16 h) clustered together. The highest number of metabolites detected from the global metabolomic profile were derived from the amino acid metabolic super pathway at 155 metabolites, followed by the nucleotide (48), lipid (41), and carbohydrate (29) super pathways. Hierarchical clustering was then performed on the top 50 significant (*p* ≤ 0.05) highest detected metabolites compared with the control. Clustering was similar across the four strains (Figure 3A) for the 50 metabolites. To further analyze how the strains differ based on their metabolic profiles, a principal component analysis (PCA) and a partial least-squares discriminant analysis PLS_DA plot of the global metabolite profile values were performed (Figures 3B,C). The PCA and PLS-DA plots of the 552 metabolites detected confirmed the data in the HCA showing *L. acidophilus* and *L. gasseri* clustering together, while *L. crispatus* and *L. rhamnosus* cluster separately from *L. acidophilus* and *L. gasseri*, each indicating differential metabolic profiles for *L. crispatus* and *L. rhamnosus* from the other strains.

**Figure 4:**
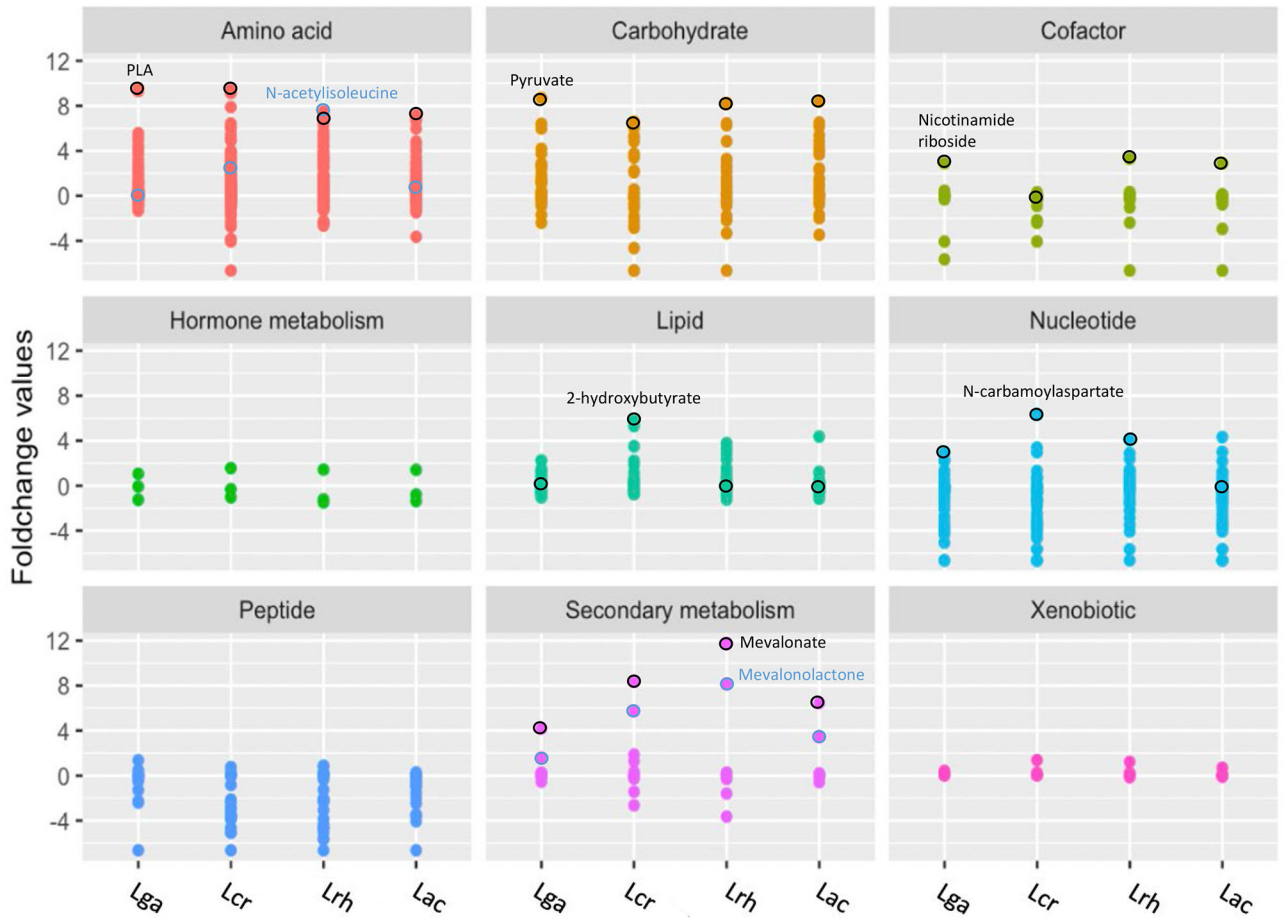
Representation of the global metabolic profile of *Lactobacillus* strains based on super pathway analysis. Significant (*p* ≤ 0.05) log2 transformed fold change values for each metabolite were used for mapping to nine biochemical pathways. Distinct colors and grids indicate the overarching biochemical pathway for the detected metabolites. Outlined circles (black and blue) indicate highly detected and unique metabolites for each strain. *L. acidophilus* NCFM (Lac), *L. crispatus* NCK1351 (Lcr)*, L. gasseri* NCK1342 (Lga), and *L. rhamnosus* GG (Lrh).

Homofermentative *Lactobacillus* metabolize monosaccharides through the Embden-Meyerhof pathway (EMP) (Goh and Klaenhammer, 2015), where glucose serves as a carbohydrate energy source that is fermented to two molecules of pyruvate. Pyruvate is often subsequently converted into lactate through NADH-dependent lactate dehydrogenase which helps maintain the redox balance by regenerating NAD^+^ (Zotta et al., 2017). Surprisingly, *L. crispatus* had a lower fold change value of pyruvate but a similar fold change value of lactate compared with the other strains. While pyruvate is mainly converted to lactate in *Lactobacillus*, it is possible that *L. crispatus* distributed pyruvate among other pathways to generate other products, such as derivatives of branched chain amino acids. Similar to the other strains, *L. crispatus* has an incomplete TCA cycle, but had a relatively higher fold change value of TCA cycle intermediates. Of the 50 metabolites with the highest fold change values, the amino acid pathway was the super pathway with the greatest number of compounds, followed by the carbohydrate, and then the lipid super pathway. Within the carbohydrate super pathway, pyruvate was the highest metabolite fold change value for all the strains (Figure 4). Interestingly, lactate was the next highest fold change value for all strains except *L. rhamnosus*, which had ribose as the second highest fold change value. *L. rhamnosus* is a facultative hetero-fermentative organism that can catabolize pentoses in addition to hexoses (Bintsis, 2018) which may explain why it had a greater fold change value of ribose.

Phenyllactate (PLA) is a compound derived from the amino acid super pathway and had the greatest increased fold change value by *L. gasseri*. Phenylpyruvate was identified in significantly smaller quantities than PLA; however, the Kyoto Encyclopedia of Genes and Genomes (KEGG) has no annotated enzymatic activities in lactobacilli to generate these products. An iterative PSI-Blast using fldH (a phenyllactate dehydrogenase from *Clostridium sporogenes* BL-8) was used to identify potential orthologs. Each species showed a 33–35% identity with potential ortholog, D-2-hydroxyacid dehydrogenase, which suggests they may contain relevant enzymatic capacity. PLA was the highest fold change value metabolite within the amino acid super pathway for all the strains except *L. rhamnosus*, which had N-acetylisoleucine as the highest fold change value followed by PLA (Figure 4). N-acetylisoleucine was a relatively minimal fold change value in the other strains.

Mevalonate is a secondary metabolite that is derived from the isoprenoid biosynthesis pathway (Kuzuyama and Seto, 2012) and is strongly related with *L. rhamnosus*. Within the secondary metabolite super pathway, all the strains had the highest fold change value of mevalonate, followed by mevalonolactone.

For the cofactor super pathway, nicotinamide riboside (NR) was the highest fold change value for all the strains except for *L. crispatus*, which did not have significantly different values than that of the control (Figure 4). Surprisingly, *L. crispatus* had biotin as the highest fold change value that achieved statistical significance; 2-hydroxybutyrate, a hydroxy fatty acid that falls within the lipid super pathway, had a significantly increased fold change value for *L. crispatus* but none of the other strains. Interestingly, *L. crispatus* does not have a complete annotated propanoate metabolism pathway in KEGG, but it does have two L-lactate dehydrogenase enzymes that are necessary for the conversion of 2-oxobutyrate to 2-hydroxybutyrate. N-carbamoylaspartate is a nucleotide derivative from pyrimidine metabolism that was the highest fold change value for all strains except *L. acidophilus*, which had no significant production of this metabolite and the highest increased fold change value of cytosine (Figure 4).

#### Specific Metabolite Detection for *Lactobacillus* Strains and Controls

**Figure 5:**
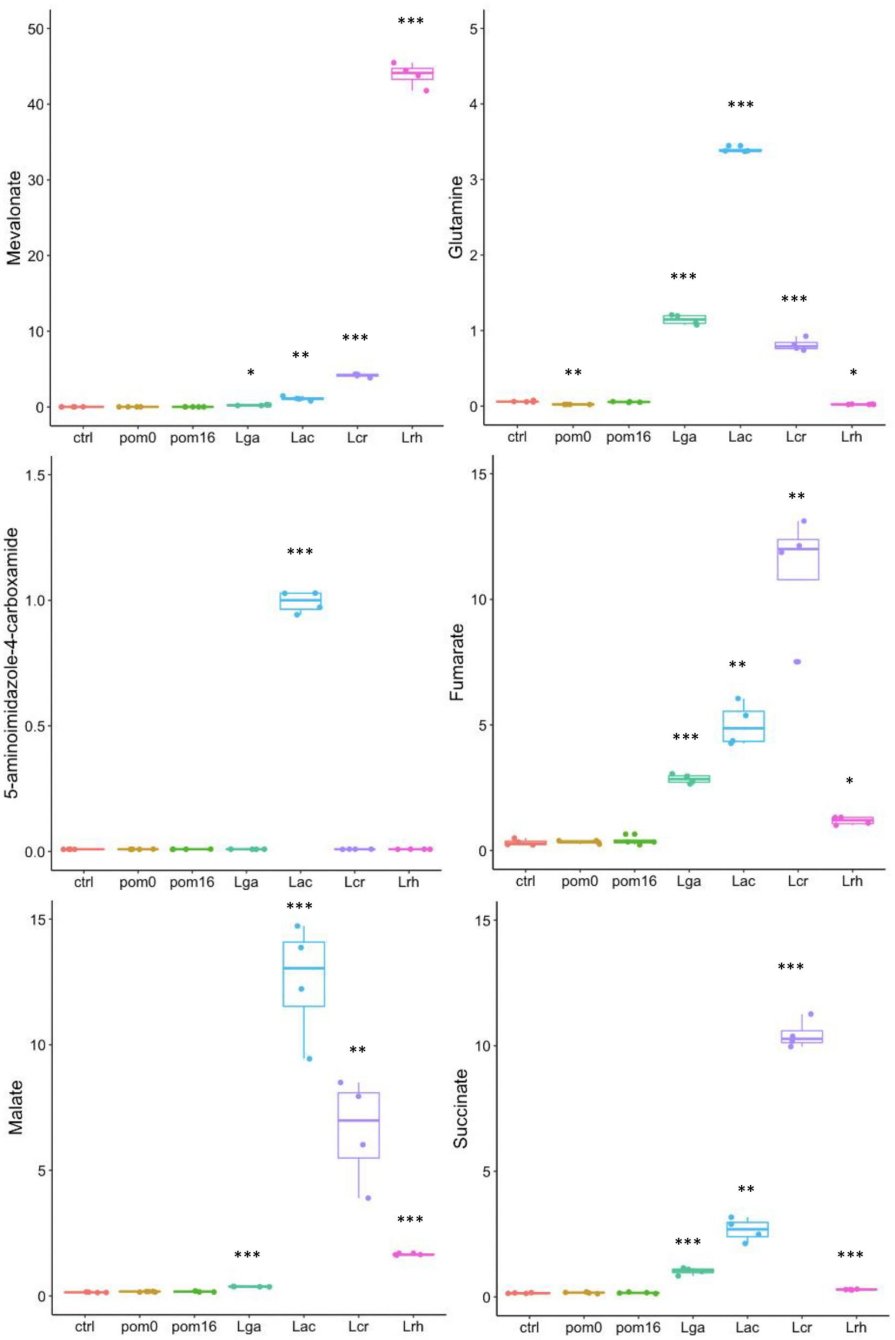
Boxplots of specific metabolites. The data points are from the metabolomic data that were normalized and scaled to the median value for each compound. Boxplots for mevalonate, glutamine, 5-aminoimidazole-4-carboxamide, fumarate, malate, and succinate are shown. *L. acidophilus* NCFM (Lac), *L. crispatus* NCK1351 (Lcr)*, L. gasseri* NCK1342 (Lga), and *L. rhamnosus* GG (Lrh), SDM media alone (ctrl), SDM media with POM 400 μg/ml at T0 (pom0) and T16 (pom16). *p*-values are indicated as follows: *p ≤ 0.05, **p ≤ 0.005, ***p ≤ 0.0005, and they were determined by comparison to the control.

In order to observe the range of the highlighted metabolites that were unique to each strain, a boxplot was created for selected individual compounds (Figure 5). The values displayed are from the metabolomic data that were normalized and scaled to the median value for each compound. Mevalonate was highly correlated with *L. rhamnosus*, and this was confirmed from the scaled values as mevalonate was produced in the greatest quantity by *L. rhamnosus* followed by *L. crispatus, L. acidophilus*, and *L. gasseri* (Figure 5). The *Lactobacillus* strains were able to produce varying amounts of TCA cycle intermediates, including fumarate, succinate, and malate. *L. crispatus* had the highest fumarate production among the strains which could presumably be attributed to the higher enzymatic activity of fumarate reductase and fumarate hydratase. Glutamine is an α-amino acid that is derived from glutamate within the amino acid superfamily. Glutamine was detected at the highest levels in *L. acidophilus*, followed by *L. gasseri, L. crispatus*, and *L. rhamnosus*. The last illustrated metabolite is also derived from the glutamate family, 5-aminoimidazole-4-carboxamide, and is an intermediate in purine biosynthesis. Interestingly, this compound was only significantly detected in the *L. acidophilus* sample but in none of the other strains (Figure 5).

#### Conserved Loci Relative to *L. acidophilus* NCFM Transcriptional Profile

**Figure 6:**
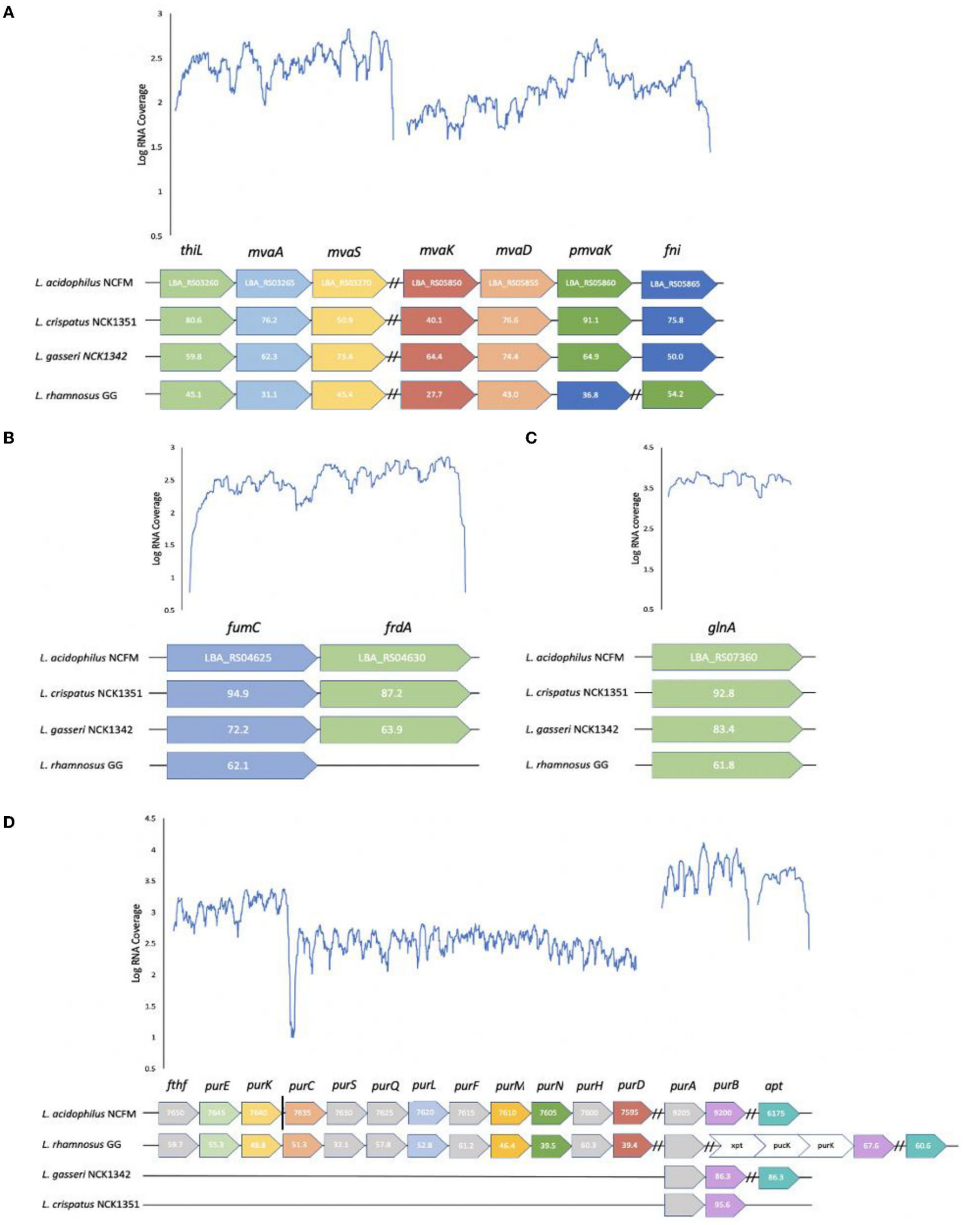
Loci comparison across strains and transcriptional profiles of *L. acidophilus* NCFM for defined loci in metabolite pathways. Conserved loci and amino acid sequence identities relative to *L. acidophilus* NCFM are shown in select *Lactobacillus* strains for **(A)** mevalonate **(B)** fumarate **(C)** glutamine, and **(D)** 5-aminoimidazole-4-carboxamide. The gray and white arrows represent genes that are not required in the specific metabolic pathway but are located within the operon. The vertical solid black line distinguishes between operons but loci are adjacent to one another on the chromosome. Dashed black lines distinguish between operons on separate parts of the chromosome.

The metabolomic analysis highlighted several unique metabolites that were associated with a specific strain. To determine whether these differences in metabolite production could be explained by variations in their biochemical pathways, a comparison of the strain's corresponding genes was analyzed. For mevalonate, the genes are split across two main loci. The first locus encodes an acetoacetyl-CoA thiolase (thiL; LBA_RS03260), hydroxymethylglutaryl-CoA synthase (mvaS; LBA_RS03270), and hydroxymethylglutaryl-CoA reductase (mvaA; LBA_RS03265); and the second locus encodes a mevalonate kinase (mvaK; LBA_RS05850), diphosphomevalonate decarboxylase (mvaD; LBA_RS05855), phosphomevalonate kinase (pmvaK; LBA_RS05860), and isopentenyl-diphosphate delta isomerase (fni; LBA_RS05865) (Figure 6A). However, in the case of *L. rhamnosus*, the *mvaK, mvaD*, and *fni* genes are in an operon and the *pmvak* gene is in its own locus.

As mentioned previously, fumarate is an intermediate compound in the TCA cycle which is incomplete in lactic acid bacteria (LAB). The active portion of the partial TCA cycle consists of two enzymes that are encoded by a fumarate hydratase (fumC; LBA_RS04630) and a fumarate reductase (frdA; LBA_RS04625) (Figure 6B); however, *L. rhamnosus* is lacking a fumarate reductase. Fumarate is produced from malate with a fumarate hydratase and from succinate with a fumarate reductase. Not surprisingly, *L. rhamnosus* produced the smallest amount of succinate and fumarate but had a higher production of malate. Of the three TCA cycle intermediates, *L. acidophilus* had the highest malate fold change, *L. crispatus* had the highest succinate fold change, and *L. gasseri* had the highest fumarate fold change.

The operon involved in the synthesis of glutamine is relatively simple compared with the other metabolites as it only encodes a glutamine synthetase (glnA; LBA_RS07360) in its own locus (Figure 6C). The difference between the strains is reflected in the fold change as *L. acidophilus* had a fold change value of 63.33, while *L. rhamnosus* had a value of only 0.42. This could perhaps reflect the potential of *L. acidophilus* having a more active glutamine synthetase and/or a more highly expressed gene.

The 5-aminoimidazole-4-carboxamide biosynthetic genes are scattered on the chromosome in four transcriptional units for *L. acidophilus* and *L. rhamnosus* (Figure 6D). The first locus is composed of formate-tetrahydrofolate ligase (fthf; LBA_RS07650), 5-(carboxyamino) imidazole ribonucleotide mutase (purE; LBA_RS07645), and 5-(carboxyamino) imidazole ribonucleotide synthase (purK; LBA_RS07640). Although *fthf* is within the operon, it is not required for the production of 5-aminoimidazole-4-carboxamide. The second locus consists of phosphoribosylaminoimidazolesuccinocarboxamide synthase (purC; LBA_RS07635), phosphoribosylformylglycinamidine (pur) synthase subunit purS (LBA_RS07630), pur synthase subunit purQ (LBA_RS07625), pur synthase subunit purL (LBA_RS07620), amidophosphoribosyltransferase (purF; LBA_RS07615), pur cyclo-ligase (purM; LBA_RS07610), phosphoribosylglycinamide formyltransferase (purN; LBA_RS07605), bifunctional AICAR transformylase/IMP cyclohydrolase (purH; LBA_RS07600), and phosphoribosylamine-glycine ligase (purD; LBA_RS07595). The third locus includes adenylosuccinate synthase (purA; LBA_RS09205) and adenylosuccinate lyase (purB; LBA_RS09200), while the fourth locus consists of an adenine phosphoribosyltransferase (aprT; LBA_RS06175) in its own operon. Genes *purS, purQ, purF, purH*, and *purA* are not involved in this metabolic pathway. Most lactobacilli are auxotrophic for both purines and pyrimidines and utilize salvage pathways in order to convert the necessary nucleobases or nucleosides to nucleotides (Kilstrup et al., 2005). 5-aminoimidazole-4-carboxamide is an intermediate in the purine metabolism pathway, and the majority of the necessary genes to produce this metabolite are found in *L. rhamnosus* and *L. acidophilus* but not in *L. gasseri* and *L. crispatus*. The *purA* and *purB* genes are conserved in all the strains in their own operon, except for *L. rhamnosus* which has *purA* in its own operon and *purB* in an operon with *xpt* (xanthine phosphoribosyltransferase), *pucK* (uric acid permease), and another *purK* gene.

## Discussion

The human gut microbiome can influence overall host health based on the microbial composition and biochemical function. *Lactobacillus* species are prominent microorganisms native to the GIT that help maintain a homeostatic environment. Attaining a balanced microbiota can be modulated with the consumption of probiotic bacteria through foods and use of probiotic supplements, which are typically composed of *Lactobacillus* and *Bifidobacterium* species. The GIT microbiota can also be positively impacted through dietary changes with the consumption of prebiotic substrates to selectively stimulate the growth of beneficial commensal microorganisms. This study aimed to investigate the metabolic profiles of four *Lactobacillus* species when grown in the presence of pomegranate extract. Some compounds previously reported as a derived metabolite from ellagitannins, including urolithins and ellagic acid, were not detected.

Evolutionary adaptions to niche environments have enabled *Lactobacillus* species to specialize and diversify their metabolic capabilities. *Lactobacillus* species are naturally found within human cavities, including the oral, gastrointestinal, and vaginal cavity. *L. acidophilus, L. rhamnosus, L. gasseri*, and *L. crispatus* species are found in all of these human environments (Klaenhammer et al., 2005; Selle and Klaenhammer, 2013; Ghosh et al., 2020). The four strains in this study were investigated to analyze two commonly used probiotics (*L. acidophilus* NCFM and *L. rhamnosus* GG) and two potential probiotics (*L. crispatus* NCK1351 and *L. gasseri* NCK1342) in combination with a polyphenolic substrate.

Lactobacilli are typically low G+C, facultative anaerobes that are fastidious in nature. From the growth assay results, when grown in SDM and SDM with POM, the strains exhibited similar growth patterns with *L. acidophilus* demonstrating a higher CFU/ml after 24 h in SDM with POM. These data show that the POM concentration of 400 μg/ml does not produce a toxic environment for these strains. To further differentiate between the strains and confirm their basic saccharolytic and enzymatic capabilities, a carbohydrate utilization and enzyme activity assay was performed. Comparative genome analyses indicate that species that adapt to specific environmental niches tend to have a simplified metabolic capacity, such as *L. gasseri*, while species from diverse niches, like *L. rhamnosus*, contain vast regulatory and transport functions (Goh and Klaenhammer, 2009; De Angelis et al., 2016). *L. acidophilus* species are commonly isolated from acidic environments and are commercially used in a variety of dairy products. *L. crispatus* and *L. gasseri* are among six predominant species that belong to the *L. acidophilus* complex based on similar metabolic and functional properties (Selle et al., 2014). *L. rhamnosus* is a ubiquitous species that has been isolated from a range of bodily habitats as well as certain fermented dairy products and has also been associated with beer spoilage (Bernardeau et al., 2008). Results from the carbohydrate utilization and enzyme API assays showed *L. rhamnosus* was able to ferment the widest range of sugars and had the broadest enzyme activity. However, *L*. rhamnosus GG was not able to utilize lactose, a common substrate forLAB, due to frameshifts in the antiterminator (*lacT*) and 6-phospho-β-galactosidase (*lacG*) genes (Kankainen et al., 2009). Glycosyl hydrolases are key enzymes of carbohydrate metabolism that include α- and β-glucosidase, α- and β-galactosidase, β-glucuronidase, α-mannosidase, α-fucosidase, and N-acetyl-β-glucosaminidase from the enzyme activity assay. All of the strains showed strong activity for the α- and β-glucosidases and β-galactosidase, and these results were expected as one of the key features of gastrointestinal lactobacilli is their prominent saccharolytic capabilities. Interestingly, *L. rhamnosus* was the only strain with α-fucosidase activity and has been reported as one of the only sequenced intestinal lactobacilli with this enzyme (Morita et al., 2009). α-fucosidases cleave biological substrates that contain fucosyl-oligosaccharides into L-fucose compounds, which have been suggested to have protective roles in the gut and systemic infection and inflammation (Pickard and Chervonsky, 2015).

Untargeted metabolomic analysis of cell-free supernatants from the four *Lactobacillus* strains offered insights into interconnected metabolic pathways and the production of unique metabolites. Phylogenetic analyses of *Lactobacillus* species have identified key genes acquired through horizontal gene transfer (HGT) and the loss of non-essential genes as the LAB coevolved with their habitats (Makarova et al., 2006; O'sullivan et al., 2009). The complete mevalonate pathway might have been acquired through HGT, and the specific organization of genes in this pathway is conserved in a single operon in most *Lactobacillales* genomes (Makarova et al., 2006). In these strains, this pattern is observed in *L. acidophilus, L. crispatus*, and *L. gasseri*; however, *L. rhamnosus* has a phosphomevalonate kinase in a separate operon. Lactobacilli are able to utilize various substrates for growth, primarily amino acids, nucleotides, carbohydrates, and lipids. From the HCA (Figure 3A), *L. acidophilus* and *L. gasseri* had the most similar metabolite profiles, followed by *L. crispatus*, and then *L. rhamnosus*. This is not surprising, as *L. gasseri* was previously classified as *L. acidophilus* up until 1980 due to similar phenotypical characteristics, but technological advances with DNA/DNA hybridization proved they are distantly related (Lauer and Kandler, 1980). The top 50 highest fold change value metabolites among the strains were selected and displayed in a heat map (Figure 3A). From these selected metabolites, most of the compounds were derived from the amino acid super pathway, followed by the carbohydrate, and then the lipid super pathway. Most *Lactobacillus* species are auxotrophic for amino acids and are incapable of synthesizing them *de novo*. They compensate for this shortcoming with various amino acid and peptide uptake systems and peptidases to effectively utilize exogenous nitrogen sources (Goh and Klaenhammer, 2009). Once LAB acquire amino acids, various enzymes such as aminotransferases and decarboxylases play an important role in these catabolic pathways (Fernández and Zúñiga, 2006). PLA was the highest fold change value metabolite within the amino acid super pathway for all the strains except *L. rhamnosus*, which had N-acetylisoleucine as the highest produced compound followed by PLA (Figure 4). PLA is converted from phenylpyruvate with a phenyllactate dehydrogenase, which has not been annotated in any of these *Lactobacillus* species. However, a protein blast with a potential ortholog from *C. sporogenes* showed that there is potential enzymatic capacity. Many other lactobacilli have the ability to convert phenylalanine to PLA (Valerio et al., 2004), which can be considered a desirable trait. Several studies have reported PLA to have inhibitory effects on several fungal species and bacterial contaminants found in humans and food products, including *Listeria monocytogenes, Staphylococcus aureus*, and *Enterococcus faecalis* (Dieuleveux and Guéguen, 1998; Dieuleveux et al., 1998; Lavermicocca et al., 2000).

Glutamine and 5-aminoimidazole-4-carboxamide are derived from glutamate and were produced the most by *L. acidophilus*. Glutamine synthetase, *glnA*, catalyzes the formation of L-glutamine by adding ammonia (NH_3_) to L-glutamate. This reaction plays a crucial role in the regulation of nitrogen metabolism (Siragusa et al., 2014). In addition, GlnA has been reported to be involved in acid stress resistance of *L. rhamnosus* GG (Koponen et al., 2012). These strains all contain a *glnA* gene, with *L. crispatus* having the most sequence similarity to *L. acidophilus*, followed by *L. gasseri* and *L. rhamnosus*. Glutamine is an amino acid found in abundance within the human body that has been shown to have an important role in intestinal physiology (Kim and Kim, 2017). The beneficial role of glutamine in the intestine has been observed by the promotion of enterocyte proliferation, regulation of tight junction proteins, and suppression of proinflammatory signaling pathways (Rhoads et al., 1997; Demarco et al., 2003; Li et al., 2004; Xue et al., 2011).

Purine nucleotides are synthesized *de novo* and result in the formation of inosine monophosphate (IMP), which then branches into two separate routes that lead to adenosine monophosphate (AMP) or guanosine monophosphate (GMP) (Kilstrup et al., 2005). Prior to IMP formation, 5-aminoimidazole-4-carboxamide can be produced in a 10-step reaction sub-pathway in purine biosynthesis. *L. acidophilus* and *L. rhamnosus* encode all of the genes necessary to produce 5-aminoimidazole-4-carboxamide in this specific pathway, while *L. gasseri* and *L. crispatus* encode two and one genes, respectively. Although *L. gasseri* and *L. crispatus* do not encode all the necessary genes to produce 5-aminoimidazole-4-carboxamide, they can still produce IMP from alternative pathways. Surprisingly, *L. gasseri* and *L. crispatus* only contain two purine biosynthesis genes, *purA* and *purB*. *purB* encodes an adenylosuccinate lyase that catalyzes the conversion of succinyl aminoimidazole carboxamide ribotide (SAICAR) into aminoimidazole carboxamide ribotide (AICAR) in the *de novo* purine synthesis pathway and the conversion of succinyl-AMP (sAMP) to AMP (Singer et al., 2016). Gene *purA* encodes an adenylosuccinate synthase and performs the reverse reaction of *purB* by converting sAMP to AMP.

As mentioned earlier, LAB are obligate fermentative organisms, and therefore, they have a partial or incomplete TCA cycle. However, the *L. acidophilus, L. gasseri*, and *L. crispatus* genomes encode fumarate reductase and fumarate hydratase that enable the production of fumarate, malate, and succinate. Fumarate reductase is a key enzyme in anaerobic respiration that uses fumarate as the terminal electron acceptor. This is a common property among gram-negative bacteria and some facultative anaerobic gram-positive bacteria (Van Hellemond and Tielens, 1994). *L. rhamnosus* lacks a fumarate reductase that would inhibit its ability to perform anaerobic respiration *via* this pathway and reduce its ability to produce fumarate.

Secondary metabolites are organic compounds that are not directly involved in normal growth, development, or reproduction but may confer a selective advantage to bacteria (Craney et al., 2013). Isoprenoids are a diverse group of secondary metabolites that have important biological functions in bacteria such as with electron transport and as cell wall biosynthesis intermediates (Kuzuyama and Seto, 2012). All isoprenoids are synthesized by the condensation of isopentenyl diphosphate (IPP) to dimethylallyl diphosphate (DMAPP). The mevalonate pathway results in the formation of IPP from acetyl-CoA and has been established mainly in mice and *Saccharomyces cerevisiae* (Kuzuyama and Seto, 2012). Interestingly, most prokaryotes lack the gene homologs of the mevalonate pathway and utilize the 2-*C*-methyl-d-erythritol 4-phosphate (MEP) pathway (Kuzuyama and Seto, 2012). However, all the *Lactobacillus* strains in this study exhibited the necessary genes for the production of IPP through the mevalonate pathway. Mevalonate was the highest produced secondary metabolite for all strains, followed by mevalonolactone, which is the dehydrated product of mevalonic acid.

The untargeted metabolomic analysis from this study offered insights into the metabolic pathways of several *Lactobacillus* strains grown in the presence of pomegranate. These species have evolved to adapt to their specific environmental niches, which has altered their metabolic capacities. However, these species are all inhabitants of the human GIT and therefore exhibit some of the same metabolic characteristics. Previous studies have identified specific metabolites derived from pomegranate ellagitannins that may have beneficial effects on the human host, such as urolithins and ellagic acid (Bialonska et al., 2009, 2010; Li et al., 2015). Although these specific compounds were not detected, many other metabolites that are unique to each strain and can contribute to the host health were observed. The production of these metabolites could enhance the probiotic effect of these strains by providing additional health benefits. Understanding specific metabolic processes is useful to mechanistically understand probiotic function in the GIT and is essential in selecting specific probiotic strains in formulation development.

## Data Availability Statement

The raw data supporting the conclusions of this article will be made available by the authors, without undue reservation.

## Author Contributions

MC performed the experiments and analyses and wrote the manuscript. SO'F designed the experiments, prepared the samples for metabolomics, and wrote the manuscript. NC designed the experiments and edited the manuscript. RB provided input on the experimental design and edited the manuscript. The data presented were part of MC's MS Thesis (https://repository.lib.ncsu.edu/handle/1840.20/39039). All authors contributed to the article and approved the submitted version.

## Funding

This work was funded by Elysium Health LLC and the North Carolina Agricultural Foundation. The funder Elysium Health LLC was not involved in the study design, collection, analysis, interpretation of data, the writing of this article or the decision to submit it for publication.

## Conflict of Interest

The authors declare that the research was conducted in the absence of any commercial or financial relationships that could be construed as a potential conflict of interest.

## Publisher's Note

All claims expressed in this article are solely those of the authors and do not necessarily represent those of their affiliated organizations, or those of the publisher, the editors and the reviewers. Any product that may be evaluated in this article, or claim that may be made by its manufacturer, is not guaranteed or endorsed by the publisher.
